# Grado de satisfacción y conocimiento de pacientes positivos para HIV ante el cambio de tenofovir a tenofovir-alafenamida en tratamientos con emtricitabina y rilpivirina

**DOI:** 10.7705/biomedica.4989

**Published:** 2020-08-20

**Authors:** Manuel Vélez-Díaz-Pallarés, Teresa Gramage-Caro, Miguel Ángel Rodríguez-Sagrado, Beatriz Montero-Llorente, Teresa Bermejo-Vicedo

**Affiliations:** 1 Servicio de Farmacia, Hospital Universitario Ramón y Cajal, Madrid, España Hospital Universitario Ramón y Cajal Madrid España

**Keywords:** VIH, tenofovir, rilpivirina, farmacéuticos, satisfacción del paciente, conocimiento de la medicación por el paciente, HIV, tenofovir, rilpivirine, pharmacists, patient satisfaction, patient medication knowledge

## Abstract

**Introducción.:**

La satisfacción y el conocimiento del cambio de tenofovir por tenofovir- alafenamida en pacientes con HIV no se han estudiado aún. Estos dos parámetros se relacionan con mejores resultados en salud y, por lo tanto, es importante medirlos durante la práctica clínica habitual.

**Objetivo.:**

Evaluar el grado de conocimiento y satisfacción de los pacientes positivos para HIV ante el cambio de tratamiento antirretroviral con rilpivirina, emtricitabina y tenofovir (RPV-FTC-TDF) por rilpivirina, emtricitabina y tenofovir-alafenamida (RPV-FTC-TAF).

**Materiales y métodos.:**

Se llevó a cabo un estudio prospectivo en un hospital de tercer nivel entre los meses de septiembre y noviembre de 2018. Se incluyeron pacientes previamente tratados con RPV-FTC-TDF que acudían por segunda vez a consulta para recibir el tratamiento con RPV-FTC-TAF. La satisfacción y el grado de conocimiento se analizaron mediante nueve preguntas, usando una escala de tipo Likert de 5 puntos para evaluar el grado de acuerdo.

**Resultados.:**

Se incluyeron 116 pacientes en el estudio. El 75 % de ellos se mostró satisfecho con el cambio y se consideró que el 64 % conocía lo que implicaba. Los pacientes jóvenes se mostraron menos satisfechos con el modo en que se les explicó el cambio (p=0,0487). Los pacientes estaban mejor informados sobre las ventajas renales (85 % de conocimiento) y óseas (82 %) de la nueva medicación, que sobre sus inconvenientes para el perfil lipídico (40 %).

**Conclusiones.:**

En general, los pacientes se mostraron satisfechos con el cambio de medicación y conocían la posología del medicamento y las ventajas de la tenofovir- alafenamida frente al tenofovir, pero no sus posibles efectos adversos.

## Abstract (en)

**Introduction:**

Satisfaction and knowledge among patients with HIV after switching from tenofovir to tenofovir/alafenamide remain unexplored. Given that both parameters are associated with better health outcomes it is relevant to measure them in patients during routine clinical practice.

**Objective:**

To evaluate the degree of knowledge and satisfaction in patients who had their antiretroviral regimen switched from rilpivirine (RPV)/emtricitabine (FTC)/TDF to RPV/FTC/TAF.

**Materials and methods:**

We conducted a prospective study in a third-level hospital between September, 2018, and November, 2018. We included patients who had previously been treated with RPV/FTC/TDF and collected their RPV/FTC/TAF treatment in the second visit. A 5-point Likert-type agreement/disagreement scale was used to assess satisfaction and knowledge regarding the medication switch.

**Results:**

We included 116 patients in the study of whom 75% were satisfied and 64% had a high-level of knowledge. Young patients were less satisfied with the way in which the change was explained (p=0.0487). Concerning the new medication, the patients were better informed about its renal (85% of them) and bone benefits (82%) than about its adverse effects on the lipid profile (40%).

**Conclusions:**

The patients were generally satisfied with the change in medication and well informed about the dosage and advantages of TAF over TDF, but less well informed about the possible adverse effects of TAF.

El tratamiento antirretroviral actual logra suprimir la replicación viral en la gran mayoría de los pacientes positivos para HIV, por lo que su esperanza de vida es similar a la de la población no infectada ^1^. En general, los esquemas actuales de medicamentos antirretrovirales presentan una tasa mínima de efectos adversos y una buena tolerancia.

Hoy día, en nuestro medio, se comercializan seis combinaciones de antirretrovirales de toma única diaria o esquema de pastilla única. Dos de ellos contienen rilpivirina (RPV), un inhibidor no nucleósido de la transcriptasa inversa de segunda generación, de excelente tolerancia y cómoda posología que, además, ha superado los problemas de resistencia de sus antecesores. Sin embargo, para que su efectividad sea la adecuada, los pacientes han de estar informados de que debe tomarse con los alimentos y evitar la combinación con inhibidores de la bomba de protones, como el omeprazol, para maximizar su absorción. Estas dos combinaciones incluyen emtricitabina (FTC) y tenofovir (TDF) o tenofovir-alafenamida (TAF) y son comercializadas como Eviplera® y Odefsey®, respectivamente.

El tenofovir es una molécula que ha formado parte de los esquemas terapéuticos de primera línea de todas las guías de tratamiento del HIV a nivel mundial, con excelentes niveles de eficacia. Sin embargo, es conocida su toxicidad renal y ósea, ya que incrementa la proteinuria y determinados marcadores renales, y disminuye la densidad mineral ósea ^2^.

Por otra parte, la tenofovir-alafenamida es un profármaco del anterior introducido recientemente en el mercado. Su metabolismo permite que los niveles en sangre del tenofovir sean mucho menores y, por lo tanto, que la toxicidad renal y la ósea disminuyan ^3^. Esta mejora en la molécula no disminuye la eficacia del tenofovir, pero dadas sus menores concentraciones plasmáticas, no ejerce tanta protección frente a un posible aumento del colesterol y los triglicéridos, tal y como se ha evidenciado en los ensayos clínicos ^4^^-^^6^. Los efectos de dicho incremento en los niveles de lípidos plasmáticos y en los resultados de salud se desconocen.

La eficacia similar de las combinaciones de RPV-FTC-TDF y RPV- FTCTAF, así como las potenciales ventajas de la tenofovir-alafenamida sobre el tenofovir en cuanto a efectos adversos, han resultado en que, en la actualidad, la tenofovir-alafenamida haya pasado a sustituir al tenofovir en muchas guías de tratamiento del HIV y, aunque a numerosos pacientes se les ha cambiado el tratamiento con tenofovir al esquema con tenofovir- alafenamida, son pocos los estudios que miden su grado de información y satisfacción frente a dicho cambio ^7^. En pacientes con una enfermedad crónica como el HIV en tratamiento a largo plazo de elevada eficacia, uno de los principales retos es conocer cuán satisfechos e informados están, pues ello se relaciona de forma directa con diversos resultados en salud ^8^.

En España, los medicamentos antirretrovirales se dispensan solamente en hospitales para garantizar su estricta prescripción y dispensación. Los servicios de farmacia de los hospitales son los responsables de la adquisición de los fármacos y de dispensarlos a los pacientes. Existen varios modelos de dispensación: desde la entrega de la medicación a través de una ventanilla por parte de un técnico o un auxiliar de farmacia, hasta aquellos que incluyen una consulta de atención farmacéutica monográfica de pacientes externos con HIV en la que farmacéuticos especialistas les hacen seguimiento fármaco- terapéutico. Estos profesionales se especializan tras cuatro años de residencia en un hospital y la aprobación de una prueba de oposición a nivel nacional.

En este contexto, el objetivo del presente estudio fue evaluar el grado de conocimiento y satisfacción ante el cambio del tratamiento antirretroviral con RPV-FTC-TDF por RPV-FTC-TAF.

## Materiales y métodos

Se llevó a cabo un estudio prospectivo en una consulta de atención farmacéutica monográfica de pacientes externos con HIV, de un hospital de tercer nivel entre los meses de septiembre y noviembre de 2018. En dicha consulta, los pacientes son atendidos por un farmacéutico especialista, quien les proporciona atención farmacéutica (información sobre el medicamento prescito y su posología, sus interacciones potenciales con otros medicamentos, la alimentación y los hábitos de vida saludables, el cumplimiento del tratamiento, los efectos adversos y consejos para prevenirlos, etc.).

Según el Servicio de Enfermedades Infecciosas, y tras la aprobación de la Comisión de Farmacia y Terapéutica del hospital, todos los tratamientos con RPV-FTC-TDF se cambiaron automáticamente por RPV-FTC-TAF. Los pacientes fueron informados de dicho cambio por su médico o farmacéutico. En el estudio se incluyeron a todos aquellos que previamente habían sido tratados con RPV-FTC-TDF y que en su segunda visita recogieron el RPV-FTC-TAF. El tamaño de la muestra de candidatos para participar en el estudio fue de 120.

En la segunda visita, el farmacéutico entrevistó al paciente mediante un cuestionario de preguntas cerradas para averiguar su grado de satisfacción y conocimiento con respecto al cambio de medicación. Además, se obtuvo la información sobre las siguientes variables sociodemográficas: sexo, edad, nacionalidad de origen, nivel educativo y situación laboral.

El cuestionario incluía nueve preguntas y se usó una escala de tipo Likert de 5 puntos para evaluar el grado de acuerdo. Se consideró “paciente satisfecho” a quien contestó “muy de acuerdo” o “de acuerdo” a las dos preguntas de satisfacción y “no satisfecho” al resto. Como “paciente conocedor” se consideró a quien contestó, por lo menos, seis de las siete preguntas referidas al conocimiento con “muy de acuerdo” o “de acuerdo”, y como “no conocedor” al resto.

Los resultados se expresaron en forma de frecuencias y porcentajes, y se recogieron en Excel 2016 de forma anonimizada. Las frecuencias se compararon mediante la prueba de ji al cuadrado o el test exacto de Fisher y, las medias, mediante la t de Student para muestras independientes. Se hizo un análisis univariado empleando la razón de probabilidades (*odds ratio,* OR) para cuantificar la asociación del conocimiento y la satisfacción con las variables del estudio (sexo, edad, nacionalidad de origen, nivel de educación, tipo de profesión y situación laboral). Los resultados se consideraron significativos si el valor de *p* era inferior a 0,05. El análisis se hizo con el programa Stata™, versión 13.

El estudio cumplió con la normativa ética vigente actualmente, así como con los procedimientos establecidos por el hospital en lo que respecta al acceso y revisión de historias clínicas. La revisión de la práctica clínica habitual se ajustó a los principios generales de la ética de la investigación en humanos establecidos en la Declaración de Helsinki.

## Resultados

Se incluyeron 116 pacientes en el estudio. Sesenta y cinco de ellos (56 %) fue informado del cambio de tratamiento por su farmacéutico, 49 (42 %) por su médico y el 2 (2 %) por ambos profesionales. Ochenta y siete se mostraron satisfechos (75 %) de los pacientes y 75 (67 %) se consideraron conocedores.

La única variable que se asoció de manera estadísticamente significativa con la satisfacción fue la edad, ya que los pacientes jóvenes se mostraron menos satisfechos con la forma en que se explicó el cambio (p=0,0487). No hubo asociación significativa entre el conocimiento y las variables.

**Figura 1:**
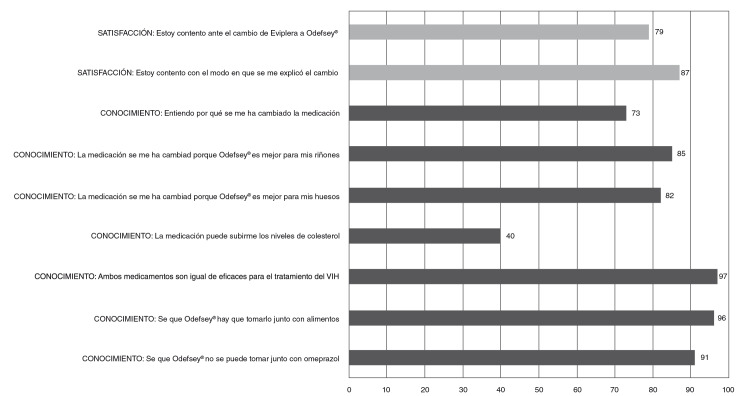
Satisfacción y conocimiento de los pacientes

En el cuadro 1 se muestran las características sociodemográficas de los pacientes y, en la figura 1, los resultados de la entrevista realizada.

**Cuadro 1:** Características de la muestra

Características	Valor	Satisfecho	Conocedor
Sexo, n (%)			
Varón	94 (81)	70 (74)	60 (64)
Mujer	22 (19)	17 (77)	15 (68)
Edad, media ± desviación estándar (años)	43,3 ± 10,4	-	-
Nacionalidad de origen, n (%)			
Española	88 (76)	66 (75)	55 (63)
América	17 (15)	14 (82)	14 (82)
Resto de Europa	8 (7)	5 (63)	5 (63)
Otros	3 (3)	2 (67)	1 (33)
Educación, n (%)			
Estudios universitarios	45 (39)	33 (73)	28 (62)
Formación profesional - bachillerato	40 (34)	29 (73)	30 (75)
Educación básica	31 (27)	25 (81)	17 (55)
Tipo de profesión, n (%)			
No sanitaria	104 (90)	76 (73)	65 (63)
Sanitaria	12 (10)	11 (92)	10 (83)
Situación laboral actual, n (%)			
Trabajador activo	91 (78)	69 (76)	61 (67)
En paro o jubilado	25 (22)	18 (72)	14 (56)

## Discusión

Los resultados evidenciaron que tres de cada cuatro pacientes estaban satisfechos con la información recibida sobre el cambio de tratamiento. Además de informarles del cambio en sus tratamientos, la intervención del farmacéutico en la atención de los pacientes positivos para HIV también ayuda a reducir los errores de medicación y prescripción de los medicamentos ^9^. En la actualidad, las funciones de estos profesionales de la salud comienzan a definirse y estandarizarse en guías ^10^.

Al igual que en otros estudios, en este llamó la atención que los pacientes jóvenes mostrasen niveles de satisfacción más bajos ^11^, lo que seguramente se debe a que tienen mayores expectativas en cuanto al sistema de salud e indicaría la necesidad de individualizar la atención farmacéutica y de estratificar los pacientes en función de distintos parámetros ^12^.

Otras variables que se han relacionado con una mayor satisfacción son la atención en hospitales públicos comparada con la de los privados, el nivel de educación (relación no significativa en el presente estudio), el lugar de residencia ^13^ y, por supuesto, la posología ^14^, aunque en el caso del presente estudio, la posología de las dos combinaciones era igual.

En este sentido, debe destacarse la importancia que están adquiriendo en la actualidad los resultados informados por los propios pacientes, con los cuales se consigue evaluar su nivel de satisfacción con sus tratamientos. La mejoría de los parámetros objetivos y la de los subjetivos de la enfermedad, estos últimos percibidos solo por los pacientes, no siempre coinciden, por lo que su opinión y la percepción de su propia salud, así como sobre sobre sus síntomas, estado vital y calidad de vida, deben considerarse en la toma de decisiones terapéuticas conjuntamente con la evaluación de su funcionamiento físico, social y psicológico. En la actualidad, los estudios clínicos ^15^^,^^16^ y otros de corte transversal ^17^ incluyen este tipo de variables.

En cuanto al grado de conocimiento, cabe destacar que las actividades educativas en los pacientes crónicos se han relacionado con mejoras en el cumplimiento del tratamiento ^18^ y, en el caso concreto del HIV, el mejor conocimiento de la enfermedad se ha relacionado con incremento en el número de CD4 y la disminución de la carga viral ^19^.

En el presente estudio, el grado de conocimiento global resultó muy elevado en lo referente a la posología del medicamento (el 96 % conocía la necesidad de tomarlo junto con alimentos y, el 91 %, de no tomarlo con inhibidores de la bomba de protones). En lo referente a la tenofovir- alafenamida, los pacientes estaban mejor informados sobre las ventajas renales (85 % de conocimiento) y óseas (82 %) que sobre los inconvenientes para el perfil lipídico (40 %), seguramente por un sesgo en la información suministrada por los clínicos, que tendría que corregirse, ya que una de las mayores demandas de los pacientes es recibir más información sobre los efectos adversos de los medicamentos que se les prescriben ^20^.

Es frecuente que se oculten parcialmente estos efectos secundarios para evitar el efecto “nocebo”, sin embargo, la cantidad y el tipo de información que debe darse al paciente sigue siendo un campo aún por explorar; como lo observaron Dekkers, *et al*. en una cohorte de pacientes positivos para HIV con dislipidemia tratados con estatinas, la mayoría de los efectos adversos adjudicados a los fármacos para el colesterol se debió al mencionado efecto “nocebo” ^21^.

Las conclusiones derivadas de una revisión sistemática evidenciaron discordancias entre los 17 estudios aleatorizados incluidos en cuanto al potencial de inducir efectos adversos en pacientes debido al exceso de información que se les había suministrado, pues en algunos se estimó que, cuanto mayor era la información, mayor el número de reacciones adversas, en tanto que en otros no ocurrió así ^22^.

Las principales limitaciones del presente estudio son haberlo llevado a cabo en un único centro y con un reducido número de pacientes, por lo que la potencia fue baja a la hora de analizar las variables. Además, no se estudiaron muchas otras variables que podrían influir en el grado de satisfacción y conocimiento de los pacientes, como la duración del tratamiento con el esquema anterior, así como el tiempo transcurrido desde el diagnóstico. No obstante, los resultados obtenidos evidencian la necesidad de estratificar la atención de los pacientes positivos para HIV, con el fin de mejorar su satisfacción y conocimiento.

En general, los pacientes se mostraron satisfechos con el cambio de medicación y demostraron un buen nivel de conocimiento sobre la posología y las ventajas de la tenofovir-alafenamida sobre el tenofovir, pero no sobre sus posibles efectos adversos, por lo que los profesionales de la salud deberemos seguir trabajando en este aspecto.

El estudio evidencia que la medición de la satisfacción y el conocimiento puede contribuir a tener mayor información de los pacientes para, así, detectar aspectos que puedan mejorar la atención farmacéutica que reciben.

## References

[1] Rodger AJ, Lodwick R, Schechter M, Deeks S, Amin J, Gilson R (2013). Mortality in well controlled HIV in the continuous antiretroviral therapy arms of the SMART and ESPRIT trials compared with the general population. AIDS.

[2] Casado JL (2016). Renal and bone toxicity with the use of tenofovir: Understanding at the end. AIDS Rev.

[3] De Clercq E (2016). Tenofovir alafenamide (TAF) as the successor of tenofovir disoproxil fumarate (TDF). Biochem Pharmacol.

[4] Gallant JE, Daar ES, Raffi F, Brinson C, Ruane P, DeJesus E (2016). Efficacy and safety of tenofovir alafenamide versus tenofovir disoproxil fumarate given as fixed-dose combinations containing emtricitabine as backbones for treatment of HIV-1 infection in virologically suppressed adults: A randomised, double-blind, active-controlled phase 3 trial. Lancet HIV.

[5] Sax PE, Wohl D, Yin MT, Post F, DeJesus E, Saag M (2015). Tenofovir alafenamide versus tenofovir disoproxil fumarate, coformulated with elvitegravir, cobicistat, and emtricitabine, for initial treatment of HIV-1 infection: Two randomised, double-blind, phase 3, non-inferiority trials. Lancet.

[6] Hagins D, Orkin C, Daar ES, Mills A, Brinson C, DeJesus E (2018). Switching to coformulated rilpivirine (RPV), emtricitabine (FTC) and tenofovir alafenamide from either RPV, FTC and tenofovir disoproxil fumarate (TDF) or efavirenz, FTC and TDF: 96-week results from two randomized clinical trials. HIV Med.

[7] Lee SS, Havens JP, Sayles HR, O’Neill JL, Podany AT, Swindells S (2018). A pharmacist-led medication switch protocol in an academic HIV clinic: Patient knowledge and satisfaction. BMC Infect Dis.

[8] Dang BN, Westbrook RA, Black WC, Rodríguez-Barradas MC, Giordano TP (2013). Examining the link between patient satisfaction and adherence to HIV care: A structural equation model. PloS One.

[9] McNicholl IR, Gandhi M, Hare CB, Greene M, Pierluissi E (2017). A pharmacist-led program to evaluate and reduce polypharmacy and potentially inappropriate prescribing in older HIV- positive patients. Pharmacotherapy.

[10] Schafer JJ, Gill TK, Sherman EM, McNicholl IR (2016). ASHP Guidelines on pharmacist involvement in HIV care. Am J Health Syst Pharm.

[11] Bowling A, Rowe G, Lambert N, Waddington M, Mahtani KR, Kenten C (2012). The measurement of patients’ expectations for health care: A review and psychometric testing of a measure of patients’ expectations. Health Technol Assess.

[12] Cantillana-Suárez M de G, Manzano-García M, Robustillo-Cortés M de LA, Morillo-Verdugo R (2018). Evaluation of HIV+ patients experience with pharmaceutical care based on AMO- methodology. Farm Hosp.

[13] Umeokonkwo CD, Aniebue PN, Onoka CA, Agu AP, Sufiyan MB, Ogbonnaya L (2018). Patients’ satisfaction with HIV and AIDS care in Anambra State, Nigeria. PLoS One.

[14] Clay PG, Yuet WC, Moecklinghoff CH, Duchesne I, Tronczyński KL, Shah S (2018). A meta- analysis comparing 48-week treatment outcomes of single and multi-tablet antiretroviral regimens for the treatment of people living with HIV. AIDS Res Ther.

[15] Murray M, Pulido F, Mills A, Ramgopal M, LeBlanc R, Jaeger H (2019). Patient-reported tolerability and acceptability of cabotegravir + rilpivirine long-acting injections for the treatment of HIV-1 infection: 96-week results from the randomized LATTE-2 study. HIV Res Clin Pract.

[16] George EC, Bucciardini R, Richert L, Dedes N, Fragola V, Nieuwkerk P (2018). Patient- reported outcomes in first-line antiretroviral therapy: Results from NEAT001/ANRS143 trial comparing darunavir/ritonavir in combination with tenofovir/emtricitabine or raltegravir. J Acquir Immune Defic Syndr.

[17] Moreno-Montoya J, Barragán AM, Martínez M, Rodríguez A, González AC (2018). Calidad de vida y percepción de apoyo social en personas con HIV en Bogotá, Colombia. Biomédica.

[18] Náfrádi L, Nakamoto K, Schulz PJ. (2017). Is patient empowerment the key to promote adherence? A systematic review of the relationship between self-efficacy, health locus of control and medication adherence. PloS One.

[19] Jones D, Cook R, Rodríguez A, Waldrop-Valverde D (2013). Personal HIV knowledge, appointment adherence and HIV outcomes. AIDS Behav.

[20] Kusch MK, Haefeli WE, Seidling HM (2018). How to meet patients’ individual needs for drug information - a scoping review. Patient Prefer Adherence.

[21] Dekkers CC, Westerink J, Hoepelman AI, Arends JE (2018). Overcoming obstacles in lipid- lowering therapy in patients with HIV - a systematic review of current evidence. AIDS Rev.

[22] Jose J, AlHajri L (2018). Potential negative impact of informing patients about medication side effects: A systematic review. Int J Clin Pharm.

